# Resilience of the resident soil microbiome to organic and inorganic amendment disturbances and to temporary bacterial invasion

**DOI:** 10.1186/s40168-018-0525-1

**Published:** 2018-08-13

**Authors:** Késia Silva Lourenço, Afnan K. A. Suleiman, A. Pijl, J. A. van Veen, H. Cantarella, E. E. Kuramae

**Affiliations:** 1 Microbial Ecology Department, Netherlands Institute of Ecology (NIOO), Droevendaalsesteeg 10, 6708 PB Wageningen, The Netherlands; 2 Soils and Environmental Resources Center, Agronomic Institute of Campinas (IAC), Av. Barão de Itapura 1481, Campinas, SP 13020-902 Brazil; 3 Institute of Biology Leiden, Leiden University, Leiden, Netherlands

**Keywords:** Seasonal variation, Microbial ecology, Sustainability, Mineral fertilizer, Vinasse, Sugarcane

## Abstract

**Background:**

Vinasse, a by-product of sugarcane ethanol production, is recycled by sugarcane plantations as a fertilizer due to its rich nutrient content. However, the impacts of the chemical and microbial composition of vinasse on soil microbiome dynamics are unknown. Here, we evaluate the recovery of the native soil microbiome after multiple disturbances caused by the application of organic vinasse residue, inorganic nitrogen, or a combination of both during the sugarcane crop-growing season (389 days). Additionally, we evaluated the resistance of the resident soil microbial community to the vinasse microbiome.

**Results:**

Vinasse applied alone or 30 days prior to N resulted in similar changes in the soil microbial community. Furthermore, the impact of the application of vinasse together with N fertilizer on the soil microbial community differed from that of N fertilizer alone. Organic vinasse is a source of microbes, nutrients, and organic matter, and the combination of these factors drove the changes in the resident soil microbial community. However, these changes were restricted to a short period of time due to the capacity of the soil community to recover. The invasive bacteria present in the vinasse microbiome were unable to survive in the soil conditions and disappeared after 31 days, with the exception of the *Acetobacteraceae* (native in the soil) and *Lactobacillaceae* families.

**Conclusion:**

Our analysis showed that the resident soil microbial community was not resistant to vinasse and inorganic N application but was highly resilient.

**Electronic supplementary material:**

The online version of this article (10.1186/s40168-018-0525-1) contains supplementary material, which is available to authorized users.

## Background

Bioethanol production using different feedstocks (e.g., sugarcane, sugarbeet, corn) produces large amounts of organic residues that can be recycled as organic fertilizers. Vinasse is a by-product of ethanol production from sugarcane. Brazil is currently the largest sugarcane ethanol producer (659.1 million tons of sugarcane annually) and generates approximately 10–15 L of vinasse for every liter of alcohol produced (27.5 billion liters of ethanol and ~ 360 billion liters of vinasse annually) [1, 2]. Vinasse is usually an acidic compost (pH 3.5–5) in the form of a dark brown slurry with a high organic content (chemical oxygen demand, 50–150 g L^−1^). To avoid discharge in rivers, alternative uses of vinasse have been explored, including as a fertilizer applied directly to sugarcane plantations [2] as a source mainly of potassium but also organic matter, nitrogen, and phosphorus. Due to the high content of potassium, the rate of application of vinasse as an organic fertilizer is based on the potential for groundwater contamination by potassium and is not sufficient to supply the total N required. Consequently, vinasse is commonly applied in combination with mineral N fertilizers in sugarcane fields in Brazil. This practice of combined application of inorganic and organic fertilizers contributes significantly to increased greenhouse gas (GHG) emissions to the atmosphere, especially nitrous oxide (N_2_O) and carbon dioxide (CO_2_), due to the high water and organic content of vinasse [3–6].

Organic fertilizers are considered more environmentally friendly than inorganic fertilizers because the former allows the nutrients produced in agricultural systems to be recycled and improves soil quality. However, the application of organic residues might disrupt the resident soil microbial community. Short- and long-term impacts of inorganic fertilization practices on soil microbial community structure have been reported [7–11]. However, few studies have evaluated the impact of organic fertilizer on the resident soil bacterial community, particularly immediately after application and throughout the plant-growing season [10, 12]. Organic fertilizers directly or indirectly cause small-scale disturbances of soil habitats due to their water content, chemical and organic components, and introduction of exogenous microbes (depending on the feedstock source) [10]. The soil microbial community is usually resistant and/or resilient to exogenous microbes and returns to the original state [10, 13]. Previous studies of sugarcane have shown that the combined application of vinasse and mineral N fertilizer can alter specific bacterial groups and favors high emissions of CO_2_-C and N_2_O-N [5, 6]. When vinasse is added a few days before or after N fertilizer as an option to decrease GHG emissions, N_2_O and CO_2_ emissions decrease compared with combined application [4], but the impact on the microbial community is unknown. In addition, no studies have considered the dynamics of the soil microbial community after vinasse application through time, the soil microbiome capacity to recover from the impact of vinasse, or the potential invasion of the resident soil microbial community by microorganisms from vinasse.

Disturbances are often classified as pulse or press depending on their duration and direct or indirect effect in the physical and chemical properties of soil [14, 15]. In general, organic and inorganic fertilizer additions are pulse disturbances, they are relatively discrete, short-term events, whereas press disturbances are long-term or continuous, such as liming, that changes the soil pH, or flooding. The soil microbes may show to be resistant or resilient to the disturbances or if they appear to be sensitive, may perform differently, or appear to be functionally redundant. Resistance is defined as the degree to which a community is insensitive to a disturbance [16], and resilience is the phenomenon that a community returns to its original composition after being disturbed [16]; commonly referred to as community recovery [15, 17]. Finally, functional redundancy refers to the property that even when the community composition is sensitive and not resilient or resistant, its functions remain similarly to the original community [16]. The functionally redundant microbial community is related to the presence of functionally redundant species in the community. Thus, depending on the disturbance, duration, and microbial community stability, the community’s response can differ substantially. Given the crucial importance of maintaining soil functions, the response of soil ecosystems to disturbances (organic and inorganic fertilizers and seasonality) must be elucidated.

**Fig. 1:**
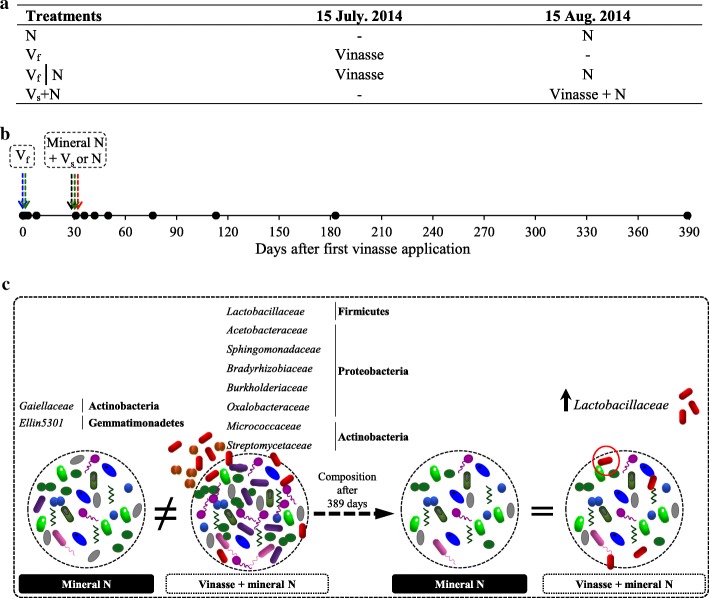
Time of application of mineral fertilizer (N: ammonium nitrate) and vinasse to sugarcane (**a** and **b**) and summary of main findings of this study (**c**). The treatments were as follows: *V*_f_, vinasse applied at day 0; N, inorganic fertilizer ammonium nitrate applied at day 30; *V*_f_│N, vinasse applied at day 0 and ammonium nitrate applied at day 30; and *V*_s_+N, vinasse plus ammonium nitrate applied only at day 30. The black dots represent the different sampling time points, and the colors of the arrows represent the different treatments: N, black; *V*_f_, blue; *V*_f_│N, green; and *V*_s_+N, red

In this study, we evaluated the recovery of the native soil microbiome after (i) multiple pulse disturbances caused by the application of organic vinasse residue, inorganic nitrogen, or both throughout the sugarcane crop-growing season (Fig. 1) and (ii) the introduction of the residue-inhabiting microbiome to the soil. The experiment was conducted under field conditions for 389 days (covering dry and rainy season) using the management practices of sugarcane farmers in Brazil. This research uses 16S rRNA gene amplicon sequencing to assess changes in the resident soil microbial community over time after the application of vinasse, mineral N, or their combined application in association with seasonal effects. In details, the consecutive multiple disturbances are the application of vinasse, representing the first disturbance, and mineral N addition after 30 days of experiment, indicating the second disturbance. Our comprehensive 16S rRNA gene sequences analysis disclosed that the application of vinasse on the same day or 30 days before N application resulted in similar changes in the soil microbial community. Furthermore, we found that the soil microbial community was more responsive to organic and inorganic fertilizers than fluctuations in seasonal temperature and rainfall through the year. Vinasse application introduced exogenous microbes that were mostly unable to persist in the soil conditions. The resident soil microbiome was not resistant to vinasse and inorganic N application but was highly resilient (Fig. 1).

## Results

### Soil microbial diversity and composition

After quality filtering, a total of 1,911,455 high-quality 16S rRNA gene sequences with an average depth of 15,170 reads per sample clustered into 8178 OTUs remained for community analysis. Comprehensive sampling of the bacterial community diversity was obtained for all treatments, with average sequence coverage of 99% determined by Good’s coverage estimator.

The treatments had no effect on the Chao1 index, with similar values between treatments and days (Additional file 1: Table S1). At days 1 and 31, application of vinasse (*V*_f_) had no effect on the alpha-diversity of the soil. However, at days 36 and 42 (5 and 11 days after mineral N fertilization), the treatments with combined application of vinasse and mineral N (*V*_f_│N and *V*_s_+N) had higher soil microbial alpha-diversity than the treatments with mineral N or first vinasse (*V*_f_) (high Simpson and Shannon index). However, after 113 days (representing rainy season), neither treatment nor seasonal climatic variation had an effect on the soil microbial alpha-diversity.

There was a consistently higher abundance of bacterial (97.35%) than archaeal (2.65%) 16S rRNA gene fragment sequences across treatments and days. In general, 29 bacterial phyla were identified, including eight major phyla: Proteobacteria (28.0% ± 3.5), Acidobacteria (19.0% ± 3.4), Actinobacteria (15.9% ± 3.0), Chloroflexi (12.5% ± 2.8), Planctomycetes (6.2% ± 1.74), Verrucomicrobia (4.9% ± 1.2), Gemmatimonadetes (3.0% ± 0.8), and Bacteroidetes (2.9% ± 1.0). The abundances of the other bacterial phyla were < 7.6%. The dominant Archaea phyla in the soils was Crenarchaeota (2.6% ± 1.3) (Additional file 1: Figure S1 and Table S2).

### Impact of multiple pulse disturbances on the soil microbial community over time

PCoA based on Bray-Curtis dissimilarity (Additional file 1: Figure S2) at a similarity cutoff of 97% at the family level showed that the soil microbial beta diversity changed during the experiment. On day 36 of the experiment (5 days after mineral N and V_s_ application), the microbial communities of the soils fertilized with vinasse on day 30 plus N fertilizer (*V*_s_+N) and with vinasse applied at day 0 and N applied 30 days later (*V*_f_│N) differed from those that received either only vinasse at day 0 (*V*_f_) or only N fertilizer at day 30 (Additional file 1: Figure S2). These changes explain the differences in the alpha-diversity analysis (Simpson and Shannon, Additional file 1: Table S1) between the treatments with combined application of vinasse and N (*V*_f_│N and *V*_s_+N) and the treatments with mineral N or *V*_f_ alone (Additional file 1: Figure S2). The effect of fertilization explained the variation in community structure until day 50 after *V*_f_ vinasse application. This dissimilarity between treatments continued to decrease at each sampling time, and the microbial communities ultimately became similar after 113 days, suggesting long-term stability of the bacterial community on the time scale of 1 year.

To more clearly asses the factors responsible for the changes in soil microbiome and their similarity among treatments, permutational multivariate analysis of variance (PERMANOVA) (*P* = 0.04) and analysis of similarity (ANOSIM) (*P* = 0.00) statistics were used due to the homogeneity of multivariate dispersions within the groups (PERMDISP *P* = 0.10 and *P* = 0.11). Treatment, day, and their interaction were the forces structuring the microbial community, with pseudo-F values of 2.21, 1.95, and 1.61 (*P* ≤ 0.04), respectively. The Pseudo-F test compares the variance of the samples; Pseudo-F higher than 1 indicates that samples are effectively different.

**Fig. 2:**
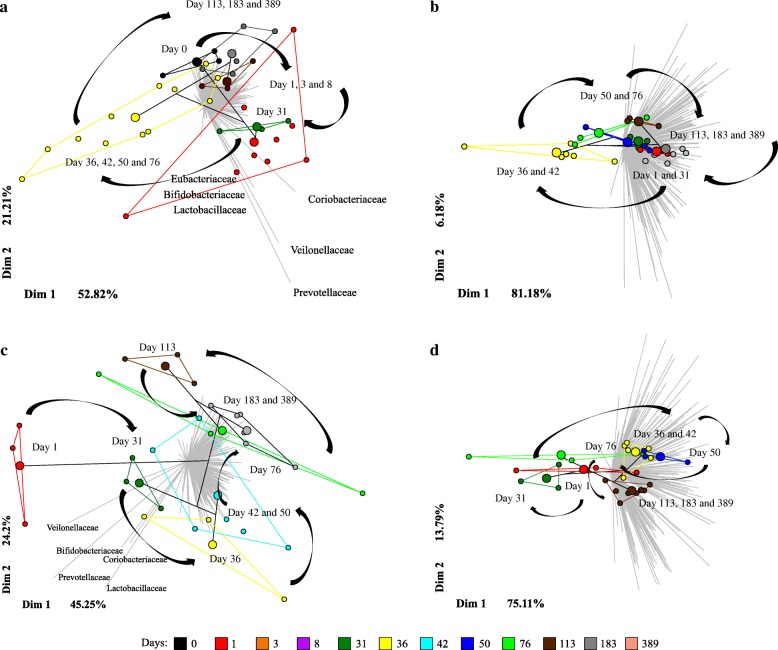
Cyclical community composition dynamics after vinasse and N fertilizer application. Multivariate regression tree (MRT) analysis was used to estimate the impact of time on bacterial community structure independently for each treatment. Six (**a**, **d**) and seven (**b**, **c**) different leaves (large colored circles) were defined based on microbial abundance and composition. The community composition within leaves is represented in a principal coordinate analysis (PCoA) plot, where small points represent individual samples and large points represent the group mean (within the leaf). The gray barplot in the background indicates families whose differential abundance explains the variation in the PCoA plot. The treatments were as follows: **a** *V*_f_, vinasse applied at day 0; **b** N, inorganic fertilizer ammonium nitrate applied at day 30; **c** *V*_f_│N, vinasse applied at day 0 and ammonium nitrate applied at day 30; and **d** *V*_s_+N, vinasse plus ammonium nitrate applied only at day 30

To further explore temporal signals in the data for different treatments, we used a multivariate regression tree (MRT) approach. The PCoA ordination given by MRT analysis showed that the microbial community dynamics appeared to be cyclical (Fig. 2), with a return to approximately the same composition after disturbance in all treatments except *V*_f_│N (Fig. 2c).

### Taxa associated with abiotic factors

To explore the biological factors involved in the differences in microbial communities between treatments, we identified taxonomic biomarkers at the family level on the days that had the highest microbial diversity and dissimilarity (days 36 and 42). Based on linear discriminant analysis effect size (LEfSe), the most enriched families in the soil were found mainly in treatments with vinasse (*V*_f_│N and *V*_s_+N) (Additional file 1: Figure S3). The top five biomarkers were Acetobacteraceae, Lactobacillaceae, Gaiellaceae, FFCH4570, and Micrococcaceae on day 36 and Dolo_23, Micrococcaceae, Burkholderiaceae, Lactobacillaceae, and Oxalobacteraceae on day 42.

### Weather conditions, soil analysis, and CO_2_ emissions

The climatic conditions during the experimental period are shown in Additional file 1: Figure S4A. The mean air temperature was 21.96 °C, with minimum and maximum air temperatures of 3.4 and 39.1 °C, respectively. Over the 389 days of the study, the cumulative rain was approximately 1064 mm (July 14 to August 15). The average of water-filled pore space (WFPS) was 66% on the sampling days (range from 60 to 94% WFPS). Part of the mineral N applied in the field area was available in mineral form (NH_4_^+^-N and NO_3_^−^-N) for approximately 80 days (Additional file 1: Figure S5). Mineral N was applied on top of the straw and after vinasse application. The pH values were similar between treatments through time (Additional file 1: Figure S5).

The microbial activity measured by CO_2_ emissions was high after vinasse application. However, CO_2_ emissions were highest in the treatment with combined application of vinasse plus N (*V*_s_+N) applied on the same day, nearly 18 g C m^−2^d^−1^. The N fertilizer treatment had the lowest CO_2_ emissions (Additional file 1: Figure S4B). However, CO_2_-C emissions increased through time with rain events and increasing temperature. Microbial activity was lower in the dry period (days 0 and 389) than in the rainy period (days 113 and 183).

**Fig. 3:**
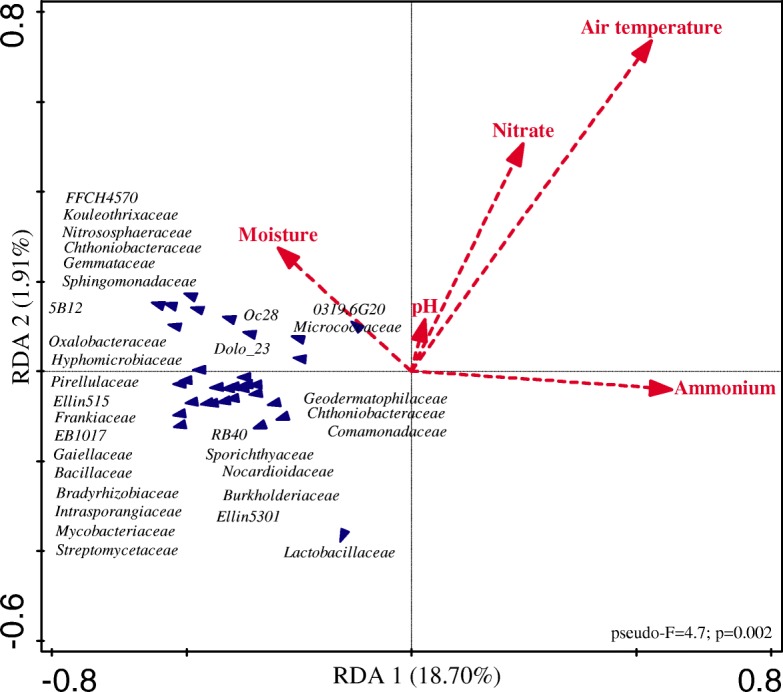
Redundancy analysis of environmental factors and the microbial community in all treatments. The treatments were as follows: *V*_f_, vinasse applied at day 0; N, inorganic fertilizer ammonium nitrate applied at day 30; *V*_f_│N, vinasse applied at day 0 and ammonium nitrate applied at day 30; and V_s_+N, vinasse plus ammonium nitrate applied only at day 30

Among all environmental factors, weather conditions, soil characteristics, and nutrient availability, soil moisture was the explanatory factor that most explained the microbial community changes in soil with vinasse, N and combined N and vinasse application (Fig. 3; pseudo-F = 4.7, *P* = 0.002). High soil moisture and low ammonium explained ~ 21.7% of the microbial community variation (axis 1, 18.70%; axis 2, 1.91%), suggesting that unmeasured biotic or abiotic factors explain the remaining ~ 78.3% of the variation.

### Effect of the vinasse microbiome on the soil microbial community

Because the two vinasses used in this study were from different batches from the same sugar mill, we assessed the microbial community composition of the *V*_f_ and *V*_s_ vinasses and determined the impact of the vinasse microbiome on the dynamics of the soil resident bacterial community after vinasse application. We then tracked back the vinasse-exogenous microorganisms using the *V*_f_ treatment.

The alpha-diversities of the two vinasses input (*V*_f_ and *V*_s_) had similar Chao1 indices. The Simpson and Shannon indices were higher in *V*_f_ than *V*_s_ (Additional file 1: Table S3). The main families found in the vinasses were Veillonellaceae, Lactobacillaceae, and Eubacteriaceae from the phylum Firmicutes (93.5% ± 4.1), Bifidobacteriaceae and Coriobacteriaceae from Actinobacteria (3.8% ± 3.6), Prevotellaceae from Bacteroidetes (2.1% ± 0.9), and Acetobacteraceae from Proteobacteria (0.4% ± 0.3). Despite the similar microbial diversity, the *V*_s_ vinasse was dominated by a single bacterial family (Additional file 1: Figure S6). The greatest difference between the vinasses was the dominance of *Megasphaera* (79.3% ± 2.1) from the family Veillonellaceae in *V*_f_ and *Lactobacillus* (96.5% ± 0.4) from Lactobacillaceae in *V*_s_; however, both of these families belong to the phylum Firmicutes. No archaeal sequences were detected in the vinasse samples (Additional file 1: Figure S6). To assess the changes, dynamics, and resilience of the soil bacterial community after vinasse-microbiome application, samples were obtained at 12 time points, including soil samples without fertilizer.

The application of vinasse to the soil altered the resident soil microbial community (Fig. 2a and Additional file 1: Figure S7). However, the difference in community composition could not be assessed by PERMANOVA because the invasive bacteria found in the vinasse caused high dispersion (PERMDISP *P* = 0.04). This was solved by removing the vinasse input counts and re-normalizing the OTU table (PERMDISP *P* = 0.20). The effect of vinasse application on the resident soil microbial community was confirmed by PERMANOVA and ANOSIM with a pseudo-F value of 1.48 (*P* < 0.04) and an R value of 0.20 (*P* = 0.00), respectively. To better visualize the effects of vinasse and environment (seasonality) on the resident soil microbial community, the PCoA was split into two figures, Additional file 1: Figures S7A and 7B. According to the Bray-Curtis dissimilarity after 1 day, the microbial community in soil fertilized with vinasse differed from that of unfertilized soil (also considered as day zero) (Additional file 1: Figure S7A). The dissimilarity continued to increase at each sampling time until day 8 and differed from day zero until day 31 (Additional file 1: Figure S7A). Finally, after 36 days, the microbial community recovered to the original state and remained stable until day 76 (Additional file 1: Figure S7B). The soil microbial community subsequently changed due to increases in temperature and soil moisture, with frequent rainy events (Additional file 1: Figure S7B).

To more clearly track the changes in microbial community composition over time scales of days throughout the year, we used an MRT approach (Fig. 2a). Consistent with the Bray-Curtis dissimilarity (Additional file 1: Figure S7), the microbial community changes through time revealed resilience. Days after vinasse application most explained the community variation (*R*^2^ = 0.303). The PCoA ordination based on MRT (Fig. 2a) showed that the microbial community dynamics appeared to be cyclical, with a return to approximately the same compositional stage as day zero after 36 days. To determine if the variation observed during the year was driven by the vinasse-exogenous microorganisms, the MRT analyses were performed again after removing all microbial sequences also found in vinasse. A similar MRT result was obtained (*R*^2^ = 0.34).

**Fig. 4:**
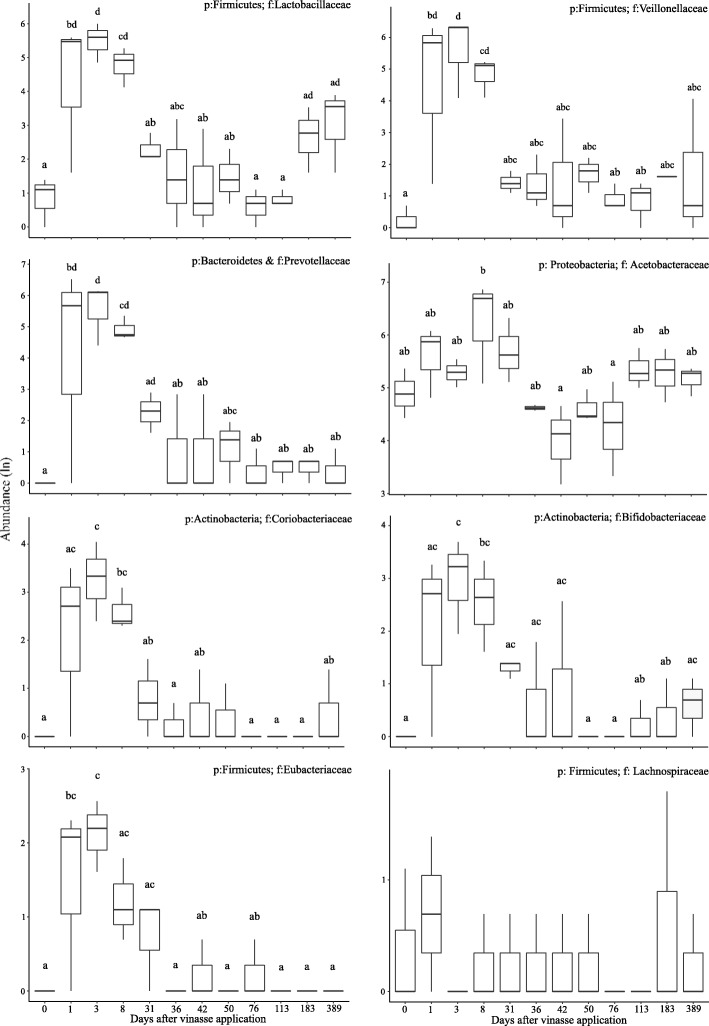
Relative abundance of bacterial families (families found in pure vinasse) in the soil after the first vinasse application. The abundances (Natural logarithm–ln of relative abundance) of the phyla (p:) and families (f:) in three replicates per day were used. Different letters indicate significant differences between days by Tukey’s HSD test (Tukey, *p* ≤ 0.05)

The LEfSe analyses showed that the relative abundances of the Lactobacillaceae, Prevotellaceae, Veillonellaceae, Micrococcaceae, Hyphomicrobiaceae, Bacillaceae, and Nitrospiraceae families changed significantly after vinasse application in the soil (Additional file 1: Table S43). The exogenous microorganisms found in vinasse were subsequently tracked in the soil samples. The main exogenous families disappeared or returned to the original state after 31 days (Fig. 4). The highest abundances of all bacteria found in vinasse were observed on day 3. The most abundant families were Lactobacillaceae, Veillonellaceae and Prevotellaceae; however, these families decreased from 266, 391, and 328 OTU counts to less than seven OTUs on day 36. Surprisingly, the relative abundance of the Lactobacillaceae family increased after 183 days (Additional file 1: Figure S8).

For the vinasse-only treatment (*V*_f_), RDA showed that nitrate concentration (NO_3_^−^-N) was the best explanatory environmental variable for soil microbial community change (Additional file 1: Figure S9; pseudo-F = 2.8, *P* = 0.002). Nitrate concentration explained ~ 36.6% of the microbial community variation (axis 1, 31.7%; axis 2, 3.80%).

## Discussion

In this study, the resident soil microbial community was highly resilient but not resistant to disturbances caused by the application of vinasse alone or in combination with N fertilizer. Vinasse is an organic residue rich in organic C, organic N, potassium, and exogenous microbes [18], and when applied to soil, it increases the soil pH, cation exchange capacity (CEC), nutrient availability, and water retention and improves soil structure [19–22]. In response to the chemical changes in soils due to vinasse addition, soil microbes with a copiotrophic lifestyle increase their abundances and activities [5, 10]. In the present study, we showed that the application of vinasse resulted mainly in increased abundances of the Bacillaceae, Micrococcaceae (Actinobacteria), Hyphomicrobiaceae, and Nitrospiraceae families. Members of Bacillaceae have been described to be mostly aerobic or facultatively anaerobic heterotrophs that grow rapidly in response to available organic C such as that found in vinasse [6, 23], while members of the phylum Actinobacteria are considered to adapt to nutrient-rich soils [5]. Surprisingly, Hyphomicrobiaceae from Alphaproteobacteria and Nitrospiraceae from Nitrospirae were the families that increased the most in soil after vinasse application. Many species of Hyphomicrobiaceae are oligocarbophilic and chemoheterotrophs that thrive only in the presence of low concentrations of suitable carbon sources and are unable to grow in rich media. However, these organisms are capable of using NO_3_^−^-N as a source of N. By contrast, *Nitrospiraceae* is highly physiologically diverse and includes chemolithoautotrophic aerobic nitrite-oxidizing bacteria that can use N from vinasse and straw mineralization [5, 6, 24]. Therefore, the nitrogen input from vinasse probably explains the increase in the abundances of Hyphomicrobiaceae [25] and Nitrospiraceae.

The application of vinasse and N fertilization alone or in combination had different effects on the soil microbial community. However, application of vinasse on the same day (*V*_s_+N) or 30 days before N application (*V*_f_│N) resulted in similar changes in the microbial community. The initial disturbance with vinasse was able to affect the stability of soil microorganisms. As a result, the already stressed microbial community was more susceptible to be affected by a second disturbance with the N addition. Apparently, the time between the *V*_f_ and N applications was not sufficient to allow significant C decomposition and N mineralization from vinasse [26, 27]. The presence of organic C after 30 days and subsequent application of N fertilizer decreases the C:N ratio and could stimulate the fast-growing microbes. However, the soil microbial communities were resilient and after 76 days the dissimilarity between the communities decreased. After 4 months, the soil communities were similar in all treatments.

The vinasse-exogenous microbes were not detected in soil after 31 days, with the exception of the families Acetobacteraceae (found in the natural soil) and Lactobacillaceae. Pitombo et al. [6] also observed an increase in the abundance of Lactobacillaceae in treatments with vinasse, but after 14 days, the relative abundance decreased and was similar to that in treatments without vinasse. However, the authors evaluated the microbial community for only a short period (46 days). Although the resident soil microbial community was resilient and returned to the original state 36 days after vinasse application (*V*_f_), an increase in the relative abundance of Lactobacillaceae was observed in all treatments with vinasse (*V*_f_, *V*_f_│N, *V*_s_+N) during the rainy period of the year (days 113 and 183) that persisted in the soil even after 1 year. *Lactobacillus* are generally aero-tolerant or anaerobic [28, 29], found in rich habitats with carbohydrate-containing substrates, besides they are the main contaminants of bioethanol production from sugarcane [28, 30]. Notably, no vinasse was applied in the experimental area previously. The straw on top of the soil throughout the experiment likely enabled *Lactobacillus* survival due to the availability of labile organic C (straw mineralization) and higher moisture content (straw retention) [31, 32]. This study is the first to show the persistence of invasive vinasse-exogenous bacteria in soil, and further studies elucidating persistence and ecological functions in soils are needed.

The soil microbial community variation was cyclical in all treatments, with small variations over time after recovery from the disturbance caused by vinasse and mineral N. Therefore, the soil microbial community was more responsive to organic and inorganic fertilizers than fluctuations in seasonal temperature and rainfall through the sampled year. The high amount of sugarcane straw (16 Mg ha^−1^) on top of the soil in the beginning of the experiment may have functioned as a barrier to water loss and soil temperature variation [31]. This barrier effect may be responsible for the small difference in the soil microbial community between the dry and rainy seasons (dry season: days 0 and 389; rainy season: day 183).

The interpretation of our results for the impacts of vinasse and vinasse-exogenous microbes on the soil resident community is subject to methodological limitations. First, the exogenous microbes present in vinasse and later found in the soil were considered invasive bacteria. By definition, a microbial invader is a microbe that was not part of the resident community prior to the time point of observation [33]. We did not use specific primers or label the vinasse-exogenous microbes to track them back in the soil. Instead, we used the number of reads in the 16S rRNA gene datasets for vinasse and for the soil samples. The microbes from vinasse were not found in the soil before vinasse application, with the exception of the Acetobacteraceae and Lactobacillaceae families. The average observed number of reads for these two families was 142 and 2, respectively, and an observation of two reads could represent a mistake during sequencing. Second, the OTU data were compositional [34]. Removing OTUs does not remove their influence on other OTUs because of the dependent structure of compositional data [34, 35]. This dependence could explain why there were no apparent differences in soil community diversity after removing bacterial families found in the vinasse community. The removal of reads is analogous to the common practice of removing eukaryotic or archaeal reads from 16S rRNA gene data. Removing reads creates a bias in the remaining data; however, the same bias is likely introduced for all days of sampling, and thus sample comparisons should remain valid. A similar approach was used by Tromas et al. [36] to predict cyanobacterial blooms in lakes.

## Conclusion

In conclusion, this study reveals soil bacterial community dynamics in response to the application of organic and/or inorganic fertilizers along the sugarcane cycle. Organic vinasse fertilizer was the main driver of changes in microbial community structure, and the soil resident communities were not resistant to vinasse application but were highly resilient. The invasive bacteria found in the vinasse microbiome were unable to survive in the soil conditions and disappeared after 31 days, with the exception of the Acetobacteraceae (native in soil) and Lactobacillaceae families.

## Methods

### Experimental setup and soil sampling

The experiment was conducted in an experimental field planted with sugarcane variety RB86-7515 located at Paulista Agency for Agribusiness Technology (APTA), Piracicaba, Brazil. The soil is classified as an Oxisol soil (soil taxonomy), and the physicochemical properties [37, 38] are shown in Additional file 1: Table S5. Sugarcane can regrow up to five times after the first harvest (ratoon cycle); in the experiment, the plants were grown for the fourth time. The sugarcane was mechanically harvested, and the straw (16 Mg ha^−1^) was left on the soil. The experiment initiated in July 15, 2014, and the last sampling was in August 8, 2015, 1 day before harvest.

The experiment was conducted in a randomized block design with three replicate blocks and a total of 12 plots (4 treatments × 3 blocks). In each plot, four 8-m-long rows spaced at 1.5 m were planted with sugarcane. In each treatment, the application time of vinasse in relation to the time of mineral N fertilization differed. Vinasse was applied either 30 days before or at the same time as N fertilization. We used two vinasses from different batches from the same sugar mill and ethanol production process. The first vinasse (*V*_f_) application was performed on day zero (July 15, 2014) (Fig. 1). Nitrogen fertilizer and the second vinasse (*V*_s_) application were performed on day 30. The treatments were as follows: (1) *V*_f_: vinasse applied at day 0; (2) N: inorganic fertilizer ammonium nitrate, applied at day 30; (3) *V*_f_│N: vinasse applied at day 0 and ammonium nitrate applied at day 30; (4) *V*_s_+N: vinasse plus ammonium nitrate applied only at day 30. The treatments were chosen based on previous results for sugarcane management practices [6].

The N fertilizer rate was 100 kg ha^−1^ of ammonium nitrate. A volume of 100 m^3^ ha^−1^ of vinasse (*V*_f_ and *V*_s_) was sprayed over the entire experimental plot using a motorized pump fit with a flow regulator. This volume of vinasse corresponds to the average application rate in sugarcane plantations. The mineral fertilizer was surface-applied on a 0.2-m-wide row 0.1 m from the plant, a common practice in commercial sugarcane production. The treatments with vinasse had a higher input of N than the mineral N treatment because vinasse contains mineral and organic N. The chemical characteristics of the vinasses applied in the experiments are shown in Additional file 1: Table S6.

Soil samples (six per plot, three samples from the two central sugarcane rows of each plot) were obtained at nine time points, 1, 31, 36, 42, 50, 76, 113, 183, and 389 days after the first vinasse (*V*_f_) application. To evaluate the vinasse effect and its potential microbial invasion, two more time points (day 3 and day 8) were added to the nine time points mentioned previously. In order to test the resilience of the microbial community after first vinasse application, soil samples without vinasse or mineral N were collected at day 1 (day 0 in the analysis).

For all treatments, soil samples (0–10 cm) were collected for determination of moisture content, NO_3_^−^-N and NH_4_^+^-N concentrations, pH, and DNA extraction. Soil subsamples (30 g) were stored at − 80 °C for molecular analysis. Soil moisture was determined gravimetrically by drying the soil at 105 °C for 24 h. Soil mineral N (NH_4_^+^-N, NO_3_^−^-N) was measured with a continuous flow analytical system (FIAlab-2500 System) after extraction with 1 M KCl, and all results are expressed per gram of dry soil. The water-filled pore space (WFPS) was calculated based on the soil bulk density (1.49 g cm^−3^) and the porosity determined at the beginning of the experiment. Climatic data were obtained from a meteorological station located approximately 500 m from the experiment.

### Respiration measurement

Fluxes of CO_2_ were measured according to the method described by Soares et al. [39] using PVC static chambers with a height of 20 cm and a diameter of 30 cm. The chambers were inserted 5 cm into the soil and 10 cm from the sugarcane rows. The two openings of the chamber cap were each fit with a valve: one for gas sampling and the other for pressure equilibration. Gases were sampled with plastic syringes (60 mL of gas) at three time intervals (1, 15, and 30 min) after the chambers were closed. The samples were transferred to pre-evacuated glass vials (12 mL) and analyzed in a gas chromatograph (model GC-2014, Shimadzu Co.) with a flame ionization detector (FID; 250 °C) [40]. Before FID detection, CO_2_ was reduced to CH_4_ by a methanizer accessory coupled to the GC. The CO_2_ flux was calculated by linear interpolation of the data from the three sampling times.

CO_2_ measurements were conducted for 389 days during the experiment. Throughout the experiment, gas samples were collected in the mornings. The gases were sampled every day during the first week, three times per week for the first 4 months, and weekly or biweekly thereafter in all treatments.

### DNA extraction

Total soil DNA was extracted from 0.25 g of soil using the MoBio PowerSoil DNA Isolation Kit (MO BIO, Solana Beach, CA, USA) according to the manufacturer’s instructions. Three replicates of each vinasse batch were also used for DNA extraction. These replicates were individual samples of the same vinasses applied in the field; we considered these samples independent in the subsequent statistical analysis. Two 50-mL aliquots of each vinasse replicate were centrifuged at 10,621*g* for 10 min on a benchtop centrifuge (Sigma 2-16P) to separate the cells from the liquid, and the pellets were combined. Total DNA was extracted from the pellets with the MoBio PowerSoil kit according to the manufacturer’s instructions. Soil and vinasse DNA quantities and qualities were determined using a Qubit 2.0 fluorometer (Life Technologies, Carlsbad, CA, USA) and a NanoDrop ND-1000 spectrophotometer (NanoDrop Technologies, Montchanin, DE, USA). The extracted DNA was also visualized on a 1% (*w*/*v*) agarose gel in Tris-acetate-EDTA (TAE) buffer.

### 16S rRNA gene amplification and sequencing

The DNA extracted from the soil was used for amplification and sequencing of the 16S rRNA. Targeting the variable V4 regions (forward primer, 515F-5′-GTGCCAGCMGCCGCGGTAA-3′; reverse primer 806R-5′-GGACTACHVGGGTWTCTAAT-3′) resulted in amplicons of ~ 300–350 bp. Dual-index and Illumina sequencing adapters were attached to the V4 amplicons. After library quantification, normalization and pooling, MiSeq V3 reagent kits were used to load the samples for MiSeq sequencing. The samples were sequenced on the Illumina MiSeq System at BGI Genomics, China.

PANDASeq [41] was used to merge paired-end reads with a minimum overlap of 50 bp and a Phred score of at least 25. Sequences were converted to FASTA format and concatenated into a single file for downstream analyses. Briefly, the OTU (operational taxonomic unit) table was built using the UPARSE pipeline; reads were truncated at 200 bp and quality-filtered using a maximum expected error of 0.5. After discarding replicates and singletons, the remaining reads were assigned to OTUs with a threshold of 97% identity. The chimera removal processes were then performed using de novo mode in UCHIME [42]. Finally, bacterial and archaeal representative sequences were searched against the Greengenes 13.5 database with a confidence threshold of 80%.

### Microbial community diversity and composition

Sampling effort was estimated by Good’s coverage [43]. Alpha-diversity analyses of rarefied OTUs were calculated using QIIME software [44]. The samples were rarefied to 3267, 2864, and 2741 reads to compare the effects of vinasse on the soil microbial community, to compare the differences between treatments, and to compare vinasses, respectively. The diversity indices were Shannon, Simpson, and Chao1 [45].

To calculate the beta diversity between groups of samples (treatments or days), a non-rarefied OTU table was used to calculate non-metric Bray-Curtis dissimilarity. The Bray-Curtis dissimilarity between treatments was calculated using QIIME software and presented in a principal coordinate analysis (PCoA) to visualize the differences in bacterial community composition. Differences in community structure between treatments, time, and their interaction were tested using permutational multivariate analysis of variance (PERMANOVA) [46] and analysis of similarity (ANOSIM) [47]. PERMANOVA and ANOSIM were performed using the “vegan” package [48] in R package version 2.4-4 with 10,000 permutations and the “adonis” and “anosim” functions, respectively (R codes are in the supplementary information). The PERMANOVA and ANOSIM tests are both sensitive to dispersion, and thus, we first tested for dispersion in the data by performing an analysis of multivariate homogeneity (PERMDISP) [49] in PRIMER v7 software.

### Community composition changes over time

We used multivariate regression tree (MTR) analyses [50] in the R “mvpart” package [51, 52] with the goal of identifying the temporal variation (time) that best explained the difference in microbial community composition in each treatment. MTR analysis is particularly useful to investigate both linear and non-linear relationships between community composition and a set of explanatory variables without requiring residual normality [53]. For the analysis, the OTU table was log-transformed, and the tree was plotted after 500 cross-validations [54], avoiding overfitting. Subsequently, the function “rpart.pca” from the “mvpart” package was used to plot a PCoA of the MTR (R codes are in the supplementary information).

### Taxa–environment relationship and taxonomic biomarker analyses

The relative abundances of taxa in each treatment, environmental factors, and daily CO_2_ fluxes were checked for normal distribution of residuals by the Kolmogorov–Smirnov (KS) test, and the data were subsequently log10-transformed. The normalized data set was used for further analyses. Soil pH was transformed to H^+^:10^−pH^ before statistical analysis. Boxplots and statistical analyses were performed in R version 3.4.0.

To investigate the taxa–environment relationship, we performed a redundancy analysis (RDA) [55] with the log10-transformed OTU table. The matrices of explanatory environmental parameters (soil and air temperatures, pH, soil moisture, NH_4_^+^-N and NO_3_^−^-N) were also log-transformed due to differences in units. RDA of microorganisms that differed significantly between days or treatments was performed to determine if interactions between environmental variables better explained the changes in the bacterial community. RDA was performed using CANOCO software for Windows 5 (Biometris, Wageningen, The Netherlands).

To explore the biological factors involved in the differences between days and treatments, we identified taxonomic biomarkers at the family level. We used linear discriminant analysis effect size (LEfSe) in Microbiome Analyst [56], a web-based tool, to identify the families that were most enriched in the soil [57]. Based on the normalized relative abundance matrix, the LEfSe method uses the Kruskal–Wallis rank-sum test to detect features with significantly different abundances between the assigned taxa and performs linear discriminant analysis (LDA) to estimate the effect size of each feature. A significance level of *α* ≤ 0.05 was used for all biomarkers evaluated in this study. The relative abundances present in vinasse and in soil (vinasse-exogenous microbes) at the taxonomic level of family were compared by Tukey’s test at *P* ≤ 0.05.

## Additional file

Additional file 1: **Table S1.** Soil microbial alpha-diversity measured in nine time points. **Table S2.** Relative abundance (%) of soil microbial phyla in sugarcane soils. **Table S3.** Soil (12 time points) and vinasse (*V*_f_ and *V*_s_) microbial alpha-diversities. **Table S4.** Microbial community in family level whose abundances differed statistically by linear discriminant analysis effect size (*p* value ≤ 0.01) between days after first vinasse (*V*_f_) application in the soil. **Table S5.** Physicochemical properties parameters of soil (0 to 20 cm) (mean ± standard deviation). **Table S6.** Chemical characteristics of the different batch vinasses from first (*V*_f_) and second (*V*_s_) vinasse application to the soil. **Figure S1.** Relative abundance (%) of soil microbial phyla in sugarcane soils. **Figure S2.** Temporal changes in the soil bacterial community as depicted by Bray-Curtis dissimilarity (which accounts for changes in the relative abundance of families). **Figure S3.** Linear discriminant analysis (LDA) of statistically different family abundances between treatments at (A) day 36 and (B) day 42. **Figure S4.** (A) Rainfall, air temperature, and water-filled pore space (WFPS) and (B) total daily mean fluxes of CO_2_-C from soils with sugarcane in different treatments. **Figure S5.** (A, B) Soil mineral N (NH_4_^+^-N+NO_3_^−^-N) content (mg N kg^−1^ of dry soil) and (C) pH. **Figure S6.** First (*V*_f_) and second (*V*_s_) vinasse bacterial community composition, top 8 at family level (A). *p*: and *f*: means Phylum and Family level, respectively. **Figure S7.** (A) Temporal changes in the soil microbial community in vinasse treatment (*V*_f_) until 36 days and (B) from 42 until 389 days, as depicted by Bray-Curtis dissimilarity. Each point represents an individual sample, with colors indicating time points. The positions of the points are the average for the jackknife replicates and ellipses were drawn around the mean values to represent the interquartile range (IQR). **Figure S8.** Relative abundance of *Lactobacillaceae* family in the soil after vinasse application. **Figure S9.** Redundancy analysis of environmental and microbial community in soils with first vinasse (*V*_f_) application. (DOCX 2779 kb)
