# Supplementary material for: Resilience of the resident soil microbiome to organic and inorganic amendment disturbances and to temporary bacterial invasion
Source: Microbiome. 2018 Aug 13;6:142. doi: 10.1186/s40168-018-0525-1 (PMC6090642; doi:10.1186/s40168-018-0525-1)
Supplement: Supplementary file 1 — Table S1. Soil microbial alpha-diversity measured in nine time points. Table S2. Relative abundance (%) of soil microbial phyla in sugarcane soils. Table S3. Soil (12 time points) and vinasse (Vf and Vs) microbial alpha-diversities. Table S4. Microbial community in family level whose abundances differed statistically by linear discriminant analysis effect size (p value ≤ 0.01) between days after first vinasse (Vf) application in the soil. Table S5. Physicochemical properties parameters of soil (0 to 20 cm) (mean ± standard deviation). Table S6. Chemical characteristics of the different batch vinasses from first (Vf) and second (Vs) vinasse application to the soil. Figure S1. Relative abundance (%) of soil microbial phyla in sugarcane soils. Figure S2. Temporal changes in the soil bacterial community as depicted by Bray-Curtis dissimilarity (which accounts for changes in the relative abundance of families). Figure S3. Linear discriminant analysis (LDA) of statistically different family abundances between treatments at (A) day 36 and (B) day 42. Figure S4. (A) Rainfall, air temperature, and water-filled pore space (WFPS) and (B) total daily mean fluxes of CO2-C from soils with sugarcane in different treatments. Figure S5. (A, B) Soil mineral N (NH4+-N+NO3−-N) content (mg N kg−1 of dry soil) and (C) pH. Figure S6. First (Vf) and second (Vs) vinasse bacterial community composition, top 8 at family level (A). p: and f: means Phylum and Family level, respectively. Figure S7. (A) Temporal changes in the soil microbial community in vinasse treatment (Vf) until 36 days and (B) from 42 until 389 days, as depicted by Bray-Curtis dissimilarity. Each point represents an individual sample, with colors indicating time points. The positions of the points are the average for the jackknife replicates and ellipses were drawn around the mean values to represent the interquartile range (IQR). Figure S8. Relative abundance of Lactobacillaceae family in the soil after vinasse application. [file 40168_2018_525_MOESM1_ESM.docx]

**Resilience of the resident soil microbiome to organic and inorganic amendment disturbances and to temporary bacterial invasion**

Késia Silva Lourenço^1,2,3^, Afnan K.A. Suleiman^1^, A. Pijl^1^, J.A. van Veen^1,3^, H. Cantarella^2^, E.E. Kuramae^1^

^1^ Microbial Ecology Department, Netherlands Institute of Ecology (NIOO), Droevendaalsesteeg 10, 6708 PB Wageningen, The Netherlands

^2^ Soils and Environmental Resources Center, Agronomic Institute of Campinas (IAC), Av. Barão de Itapura 1481, 13020-902 Campinas, SP, Brazil

^3^ Institute of Biology Leiden, Leiden University, Netherlands

* Corresponding author: [E.Kuramae@nioo.knaw.nl](mailto:E.Kuramae@nioo.knaw.nl)

Keywords: Seasonal variation; microbial ecology; sustainability; mineral fertilizer; vinasse; sugarcane.

**Table S1.** Soil microbial alpha-diversity measured in nine time points. The treatments are: V_f_: vinasse applied at day 0; N: inorganic fertilizer ammonium nitrate, applied at day 30; V_f_│N: vinasse applied at day 0 and ammonium nitrate applied at day 30; and V_s_+N: vinasse plus ammonium nitrate applied only at day 30.

^a^ Symbols in the caption refer to overall ANOVA results for the given experiment.. Significant difference: ^*^ p≤ 0.05; ^**^ p≤ 0.01 and ns: Non-Significant.

^b^ Means followed by the same capital letter in the column at each treatment and lowercase letter at each day of sampling do not differ significantly by the Tukey's test (p < 0.05).

**Table S2.** Relative abundance (%) of soil microbial phyla in sugarcane soils. The treatments are: V_f_: vinasse applied at day 0; N: inorganic fertilizer ammonium nitrate, applied at day 30; V_f_│N: vinasse applied at day 0 and ammonium nitrate applied at day 30; and V_s_+N: vinasse plus ammonium nitrate applied only at day 30. The value of each bacterial group percentage is the mean of soil samples collected from three different replicates.

| Treatment | DAYS AFTER VINASSE APPLICATION | | | | | | | | | |
| --- | --- | --- | --- | --- | --- | --- | --- | --- | --- | --- |
|  | **1** | **31** | **36** | **42** | **50** | | **76** | **113** | **183** | **389** |
| *Gemmatimonadetes* | | | | | | | | | | |
|  | ns | *** (0.01) | ** (0.03) | ** (0.04) | ns | | ns | ns | ns | ns |
| N |  | 4.26 b | 1.36 a | 1.69 a |  | |  |  |  |  |
| Vs+N |  | 2.10 a | 4.28 b | 3.15 b |  | |  |  |  |  |
| Vf |  | 5.27 b | 2.56 ab | 1.92 ab |  | |  |  |  |  |
| Vf│N |  | 3.50 ab | 3.69 b | 3.16 b |  | |  |  |  |  |
| *Cyanobacteria* | | | | | | | | | | |
|  | ns | ns | ns | ** (0.03) | ns | | ns | ns | ns | ns |
| N |  |  |  | 0.49 b |  | |  |  |  |  |
| Vs+N |  |  |  | 0.11 a | |  |  |  |  |  |
| Vf |  |  |  | 0.16 a |  | |  |  |  |  |
| Vf│N |  |  |  | 0.11 a |  | |  |  |  |  |
| *Crenarchaeota* | | | | | | | | | | |
|  | ns | ns | ** (0.03) | ns | ns | | ns | ** (0.02) | ns | ns |
| N |  |  | 0.32 a |  |  | |  | 4.53 ab |  |  |
| Vs+N |  |  | 1.75 ab |  |  | |  | 7.26 b |  |  |
| Vf |  |  | 0.86 ab |  |  | |  | 2.91 a |  |  |
| Vf│N |  |  | 2.49 b |  |  | |  | 6.89 b |  |  |
| *Bacteroidetes* | | | | | | | | | | |
|  | ns | ns | ns | ns | ***(0.01) | | ns | ns | ns | ns |
| N |  |  |  |  | 2.13 a | |  |  |  |  |
| Vs+N |  |  |  |  | 4.55 c | |  |  |  |  |
| Vf |  |  |  |  | 2.73 ab | |  |  |  |  |
| Vf│N |  |  |  |  | 4.11 bc | |  |  |  |  |
| *Armatimonadetes* | | | | | | | | | | |
|  | ns | ***(0.01) | * (0.07) | ns | ns | | ns | ns | ns | ns |
| N |  | 0.38 a | 0.48 ab |  |  | |  |  |  |  |
| Vs+N |  | 0.80 b | 0.17 a |  |  | |  |  |  |  |
| Vf |  | 0.15 a | 0.74 b |  |  | |  |  |  |  |
| Vf│N |  | 0.45 ab | 0.27 ab |  |  | |  |  |  |  |
| *WS3* | | | | | | | | | | |
|  | ns | ns | ns | ns | ns | | ns | ns | ns | ns |
| N |  |  |  |  |  | |  |  |  |  |
| Vs+N |  |  |  |  |  | |  |  |  |  |
| Vf |  |  |  |  |  | |  |  |  |  |
| Vf│N |  |  |  |  |  | |  |  |  |  |
| *AD3* | | | | | | | | | | |
|  | ns | ns | ** (0.02) | ns | ns | | ns | ns | * (0.08) | ns |
| N |  |  | 0.09 a |  |  | |  |  | 0.94 a |  |
| Vs+N |  |  | 0.53 b |  |  | |  |  | 1.97 a |  |
| Vf |  |  | 0.31 ab |  |  | |  |  | 0.94 a |  |
| Vf│N |  |  | 0.60 b |  |  | |  |  | 0.88 a |  |
| *Actinobacteria* | | | | | | | | | | |
|  | ns | *** (0.00) | ***(0.00) | * (0.06) | ns | | ns | **(0.05) | ns | ns |
| N |  | 19.90 b | 11.46 a | 12.07 ab |  | |  | 15.79 ab |  |  |
| Vs+N |  | 8.48 a | 22.42 b | 18.62 ab |  | |  | 12.82 a |  |  |
| Vf |  | 22.76 b | 14.44 a | 10.58 a |  | |  | 18.09 b |  |  |
| Vf│N |  | 18.08 b | 25.36 b | 19.41 b |  | |  | 13.62 a |  |  |
| *Verrucomicrobia* | | | | | | | | | | |
|  | ns | *** (0.00) | ***(0.00) | ns | ns | | ns | ns | ns | * (0.06) |
| N |  | 4.58 a | 6.13 b |  |  | |  |  |  | 4.83 a |
| Vs+N |  | 7.74 b | 2.22 a |  |  | |  |  |  | 4.70 a |
| Vf |  | 52.99 a | 6.37 b |  |  | |  |  |  | 6.35 b |
| Vf│N |  | 5.13 a | 3.17 a |  |  | |  |  |  | 5.04 ab |
| Planctomycetes | | | | | | | | | | |
|  | ns | ***(0.01) | ***(0.00) | * (0.07) | ns | | ns | ns | ns | ns |
| N |  | 5.43 a | 10.85 b | 10.26 b |  | |  |  |  |  |
| Vs+N |  | 10.32 b | 2.10 a | 4.30 a |  | |  |  |  |  |
| Vf |  | 2.74 a | 9.80 b | 6.38 ab |  | |  |  |  |  |
| Vf│N |  | 5.56 a | 3.38 a | 5.07 ab |  | |  |  |  |  |
| Proteobacteria | | | | | | | | | | |
|  | ns | *** (0.00) | ***(0.00) | * (0.06) | * (0.07) | | **(0.05) | *** (0.00) | ns | ns |
| N |  | 30.69 b | 20.68 a | 23.48 ab | 24.87 a | | 28.01 ab | 30.47 b |  |  |
| Vs+N |  | 17.79 a | 38.12 b | 33.62 b | 40.43 b | | 37.23 b | 22.48 a |  |  |
| Vf |  | 38.18 b | 22.94 a | 18.13 a | 26.04 ab | | 23.63 a | 30.33 b |  |  |
| Vf│N |  | 30.29 b | 34.23 b | 27.66 ab | 32.80 ab | | 26.79 ab | 21.16 a |  |  |
| *Nitrospirae* | | | | | | | | | | |
|  | ns | ns | ns |  | ns | | * (0.09) | ns | ns | ns |
| N |  |  |  |  |  | | 1.29 ab |  |  |  |
| Vs+N |  |  |  |  |  | | 0.63a |  |  |  |
| Vf |  |  |  |  |  | | 2.58 b |  |  |  |
| Vf│N |  |  |  |  |  | | 1.70 ab |  |  |  |
| *Chloroflexi* | | | | | | | | | | |
|  | ns | ns | * (0.07) | ns | ns | | ns | **(0.03) | * (0.09) | * (0.09) |
| N |  |  | 10.97 a |  |  | |  | 16.16 ab | 13.56 a | 9.23 a |
| Vs+N |  |  | 8.45 a |  |  | |  | 20.74 b | 18.30 a | 11.79 a |
| Vf |  |  | 11.16 a |  |  | |  | 12.15 a | 12.85 a | 8.89 a |
| Vf│N |  |  | 9.04 a |  |  | |  | 19.47 b | 13.49 a | 11.61 a |
| *Acidobacteria* | | | | | | | | | | |
|  | ns | ***(0.01) | ***(0.00) | ns | ns | | **(0.05) | ns | ns | **(0.02) |
| N |  | 17.46 a | 33.87 b |  |  | | 23.33 ab |  |  | 15.57 a |
| Vs+N |  | 30.31 b | 9.18 a |  |  | | 13.26 a |  |  | 16.81 a |
| Vf |  | 11.40 a | 24.93 b |  |  | | 29.26 b |  |  | 20.55 b |
| Vf│N |  | 20.51 ab | 12.75 a |  |  | | 22.78 ab |  |  | 16.99 a |
| *Firmicutes* | | | | | | | | | | |
|  | * (0.09) | *** (0.00) | ***(0.00) | ***(0.00) | **(0.02) | | ns | ns | ns | ns |
| N | 0.56 a | 0.81 a | 0.52 a | 0.69 a | 1.03 a | |  |  |  |  |
| Vs+N | 0.90 a | 5.25 b | 7.07 b | 3.71 b | 1.70 b | |  |  |  |  |
| Vf | 3.46 a | 1.14 a | 1.02 a | 0.44 a | 0.86 a | |  |  |  |  |
| Vf│N | 5.24 a | 1.27 a | 1.30 a | 1.24 a | 0.96 a | |  |  |  |  |

Means followed by the same letter in the column at each treatment do not differ significantly by the Tukey's test. Significant difference: * p ≤ 0.10; ** p≤ 0.05; *** p≤ 0.01; ns: Non-significant.

**Table S3.** Soil (12 time points) and vinasse (V_f_ and V_s_) microbial alpha-diversities.

^a^ Symbols in the caption refer to overall ANOVA results for the given experiment. Difference between vinasses or days. Significant difference: ^**^ p≤ 0.05; ^***^ p≤ 0.01 and ns: Non-Significant.

^b^ Means followed by the same letter in the column at each vinasse or day of sampling do not differ significantly by the Tukey's test (p ≤ 0.05).

**Table S4.** Microbial community in family level whose abundances differed statistically by linear discriminant analysis effect size (p-value ≤ 0.01) between days after first vinasse (V_f_) application in the soil.

^a^ p: and f: means Phylum and Family level.

**Table S5.** Physicochemical properties parameters of soil (0- to 20-cm) (mean ± standard deviation).

Abbreviations are as follows: ^a^ Organic matter.

^b^ Available phosphorus, K, Ca, and Mg were extracted with ion exchange resin.

^c^ Buffer solution (pH 7.0).

^d^ CEC (Cation exchange capacity).

^e^ Soil texture determined by the densimeter method.

**Table S6.** Chemical characteristics of the different batch vinasses from first (V_f_) and second (V_s_) vinasse application to the soil.

Abbreviations are as follows: ^a^ V_f_: Vinasse applied at day zero (15 July, 2014) and V_f_: Vinasse applied at day 30 (Aug. 15, 2014).^b^ C org: Total organic carbon.

^c^ N tot: Total organic nitrogen.

^d^ NH_4_^+^-N: ammonium.

^e^ NO_3_^-^-N: nitrate.

**Figure S1.** Relative abundance (%) of soil microbial phyla in sugarcane soils. The treatments are: V_f_: vinasse applied at day 0; N: inorganic fertilizer ammonium nitrate, applied at day 30; V_f_│N: vinasse applied at day 0 and ammonium nitrate applied at day 30; and V_s_+N: vinasse plus ammonium nitrate applied only at day 30. The value of each bacterial group percentage is the mean of soil samples collected from three different replicates.


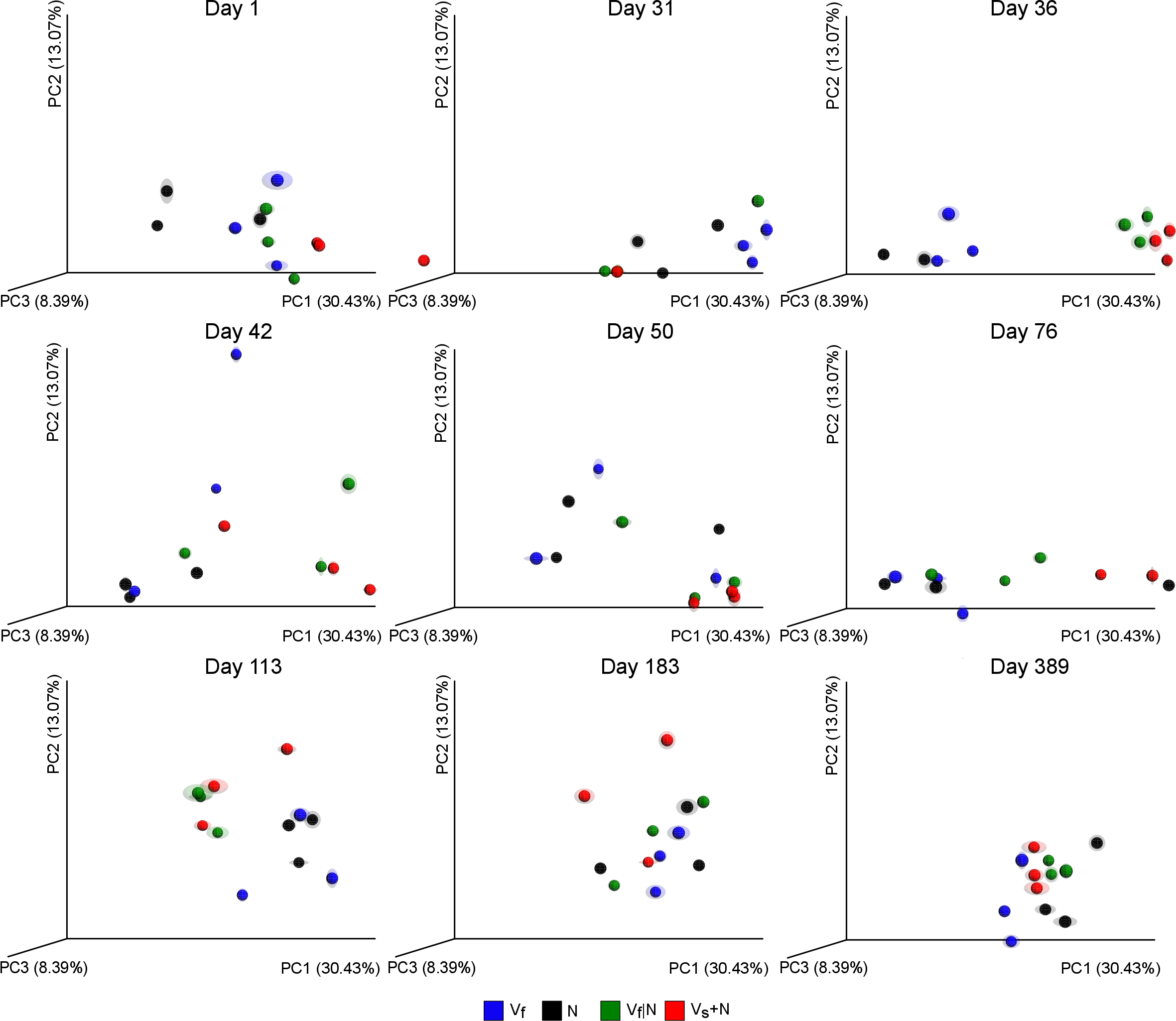


**Figure S2.** Temporal changes in the soil bacterial community as depicted by Bray-Curtis dissimilarity (which accounts for changes in the relative abundance of families). Principal coordinate analysis (PCoA) of soils cultivated with sugarcane was performed with nine time points. To illustrate the differences between treatments, each time point was showed separately for the same PCoA plot. The treatments were as follows: Vf, vinasse applied at day 0; N, inorganic fertilizer ammonium nitrate applied at day 30; Vf│N, vinasse applied at day 0 and ammonium nitrate applied at day 30; and Vs+N, vinasse plus ammonium nitrate applied only at day 30. Each point represents an individual sample, with colors indicating treatments. The positions of the points are the average for the jackknife replicates and ellipses were drawn around the mean values to represent the interquartile range (IQR).

**Figure S3.** Linear discriminant analysis (LDA) of statistically different families abundances between treatments at (A) day 36 and (B) day 42. The treatments are: V_f_: vinasse applied at day 0; N: inorganic fertilizer ammonium nitrate, applied at day 30; V_f_│N: vinasse applied at day 0 and ammonium nitrate applied at day 30; and V_s_+N: vinasse plus ammonium nitrate applied only at day 30. Significant difference: *p≤0.10; ** p≤ 0.05; and *** p≤ 0.01. f: means Family level.

**Figure S4.** (A) Rainfall, air temperature and water-filled pore space - WFPS and (B) total daily mean fluxes of CO_2_-C from soils with sugarcane in different treatments. The treatments are: V_f_: vinasse applied at day 0; N: inorganic fertilizer ammonium nitrate, applied at day 30; V_f_│N: vinasse applied at day 0 and ammonium nitrate applied at day 30; and V_s_+N: vinasse plus ammonium nitrate applied only at day 30. Vertical bars indicate the standard error of the mean (n = 3).

**Figure S5**. (A, B) Soil mineral N (NH_4_^+^-N + NO_3_^‑^-N) content (mg N kg^-1^ of dry soil) and (C) pH. The treatments are: V_f_: vinasse applied at day 0; N: inorganic fertilizer ammonium nitrate, applied at day 30; V_f_│N: vinasse applied at day 0 and ammonium nitrate applied at day 30; and V_s_+N: vinasse plus ammonium nitrate applied only at day 30.

**Figure S6.** First (V_f_) and second (V_s_) vinasse bacterial community composition, top 8 at family level (A). p: and f: means Phylum and Family level, respectivelly.


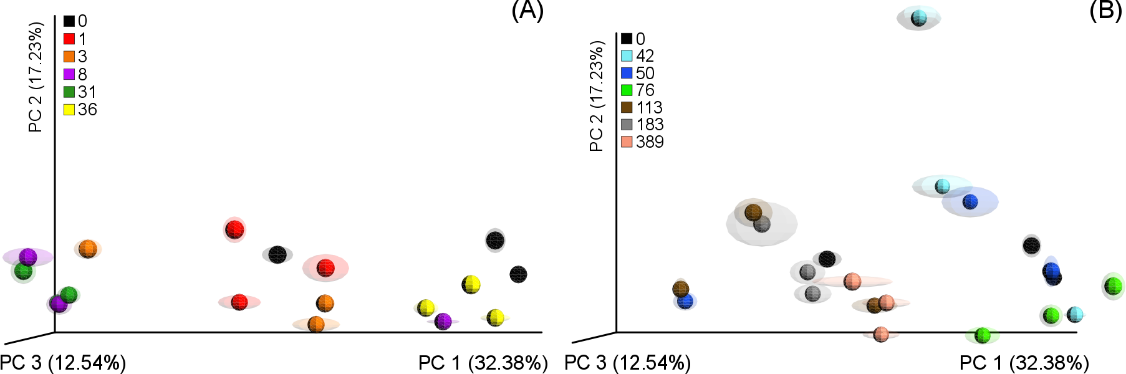


**Figure S7.** (A) Temporal changes in the soil microbial community in vinasse treatment (Vf) until 36 days and (B) from 42 until 389 days, as depicted by Bray-Curtis dissimilarity. Each point represents an individual sample, with colors indicating time points. The positions of the points are the average for the jackknife replicates and ellipses were drawn around the mean values to represent the interquartile range (IQR).

**Figure S8.** Relative abundance of *Lactobacillaceae* family in the soil after vinasse application. The abundance of three replicate/sample per day was used. The treatments are: V_f_: vinasse applied at day 0; N: inorganic fertilizer ammonium nitrate, applied at day 30; V_f_│N: vinasse applied at day 0 and ammonium nitrate applied at day 30; and V_s_+N: vinasse plus ammonium nitrate applied only at day 30.

**Figure S9.** Redundancy analysis of environmental and microbial community in soils with first vinasse (V_f_) application.

**#R libraries and scripts used in this study**

library(vegan)

library(mvpart)

library(rpart)

library(rdaTest)

library(labdsv)

library(plyr)

library(MASS)

library(phyloseq)

library(plotrix)

----------------------------------------

#Phyloseq analysis

#import biom file####

biomfile <- import_biom("otu_table.biom")

#change names (ranks to domain, phylum, etc)####

colnames(tax_table(biomfile)) <- c("Domain", "Phylum", "Class", "Order", "Family", "Genus", "Species")

#import mapping_file

biomfile_mapfile <- import_qiime_sample_data("mapping_file.txt")

#merge biomfile_prune & mapfile

biomfile_merged <- merge_phyloseq(biomfile,biomfile_mapfile)

#Permanova test using adonis function

adonis(abund_table ~ treatments*day, data=mapping_file, permutations=999)

#ANOSIM test

variable_group = get_variable(data, "variable")

variable_group = anosim(abund_table, variable_group)

----------------------------------------

#Regression_tree

time.tree <- mvpart(as.matrix(abund_table2) ~meta_table1$variable ,meta_table1,legend=FALSE, margin=0.01, cp=0, xv="pick", xval=nrow(abund_table2), xvmult=500, which=4, big.pts=T, bars=F)

rpart.pca(time.tree, interact=TRUE, colnames("none"))

----------------------------------------
